# Tanshinone IIA alleviates hypoxia/reoxygenation induced cardiomyocyte injury via lncRNA AK003290/miR-124-5p signaling

**DOI:** 10.1186/s12860-020-00264-3

**Published:** 2020-03-27

**Authors:** Liye Chen, Lili Wei, Qiongyang Yu, Haozhe Shi, George Liu

**Affiliations:** 1 Key Laboratory of Molecular Cardiovascular Science Ministry of Education; Institute of Cardiovascular Sciences, Health Science Center, Peking University, 38,Xueyuan Road,Haidian District, Beijing, 100191 People’s Republic of China; 2 School of Medicine, Shihezi University, Shihezi, 832003 People’s Republic of China

**Keywords:** Tanshinone IIA, Acute myocardial infarction, miR-124-5p, AK003290

## Abstract

**Background:**

Acute myocardial infarction (AMI) is the leading cause of death globally and has thus placed a heavy burden on healthcare. Tanshinone IIA (TSA) is a major active compound, extracted from Salvia miltiorrhiza Bunge, that possesses various pharmacological activities. The aim of the present study was to investigate the role of TSA in AMI and its underlying mechanism of action. Results: We have shown that TSA decreased the apoptosis rate, the amount of LDH, MDA as well as ROS of cardiomyocytes. Meantime, it elevated mitochondrial membrane potential (MMP) which was decreased by H/R treatment. It was also determined that miR-124-5p targets AK003290 directly. TSA up-regulated the expression of AK003290 and its function can be reversed by knock down of AK003290 as well as miR-124-5p overexpression.

**Conclusion:**

TSA exerts the protective role against H/R induced apoptosis, oxidative and MMP loss of cardiomyocytes via regulating AK003290 and miR-124-5p signaling.

## Background

Acute myocardial infarction (AMI) is characterised by a block of blood supply to the heart. It is the leading cause of death worldwide and has placed a heavy burden on healthcare [1, 2]. Restoration of blood flow, known as reperfusion, has been demonstrated to be one of the most effective therapeutic methods to prevent the heart dysfunction or damage caused by an imbalance of oxygen supply. However, reperfusion together with reoxygenation will result in an exacerbation of tissue injury as well as inflammatory responses, which is called ischemia reperfusion (I/R) injury.

Tanshinone IIA (TSA) is the major active compound present in the root extracts of Salvia miltiorrhiza Bunge, which is commonly prescribed for treating cardiovascular disease in the pharmacopoeia of China as a traditional Chinese medicine (TCM) ‘Danshen’. TSA has been shown to exert anti-angiogenic, antioxidative, anti-inflammatory, and anti-tumour activities. Increasing evidences have demonstrated the anti-tumour function of TSA in several human cancers such as the liver, cervical, gastric, colorectal, prostate, bladder, and breast cancers [3–10]. Moreover, due to its anti-oxidative and anti-inflammatory properties, TSA has been indicated to possess cardio-protective effects. For instance, the anti-oxidative function of TSA prevents atherosclerosis by reducing vascular oxidative stress, thereby inhibiting platelet aggregation and preventing endothelial damage [11–13]. Additionally, TSA pre-treatment has been shown to protect myocardium against I/R injury through the phosphatidylinositol 3-kinase/Akt-dependent pathway in diabetic rats [14].

Long non-coding RNAs (LncRNAs) are a novel group of ncRNAs with a length of more than 200 nucleotides. LncRNAs are capable of regulating gene expression at epigenetic, transcriptional and post-transcriptional levels and thus participate in various of pathological processes, such as autophagy, necrosis, and apoptosis [15].

In the present study, we focused on the function of TSA on myocardial ischemic reperfusion injury. In vitro studies revealed that TSA alleviated the apoptosis, oxidative stress and MMP loss of cardiomyocytes subjected to hypoxia/reoxygenation (H/R) treatment. In addition, I/R model of mouse was established and indicated that AK003290 was down-regulated in mouse myocardium after I/R treatment. The results of animal experiments provide a better basis for clinical transformation in the future. Mechanically, we found that TSA participates in the regulation of lncRNA AK003290/miR-124-5p signaling. These findings offer important insights into fundamental mechanisms underlying functions of TSA and lncRNAs, meantime, provided novel therapeutic targets for cardiac ischemic injury.

## Results

### TSA alleviated H/R induced apoptosis, oxidative stress and loss of mitochondrial membrane potential

**Fig. 1:**
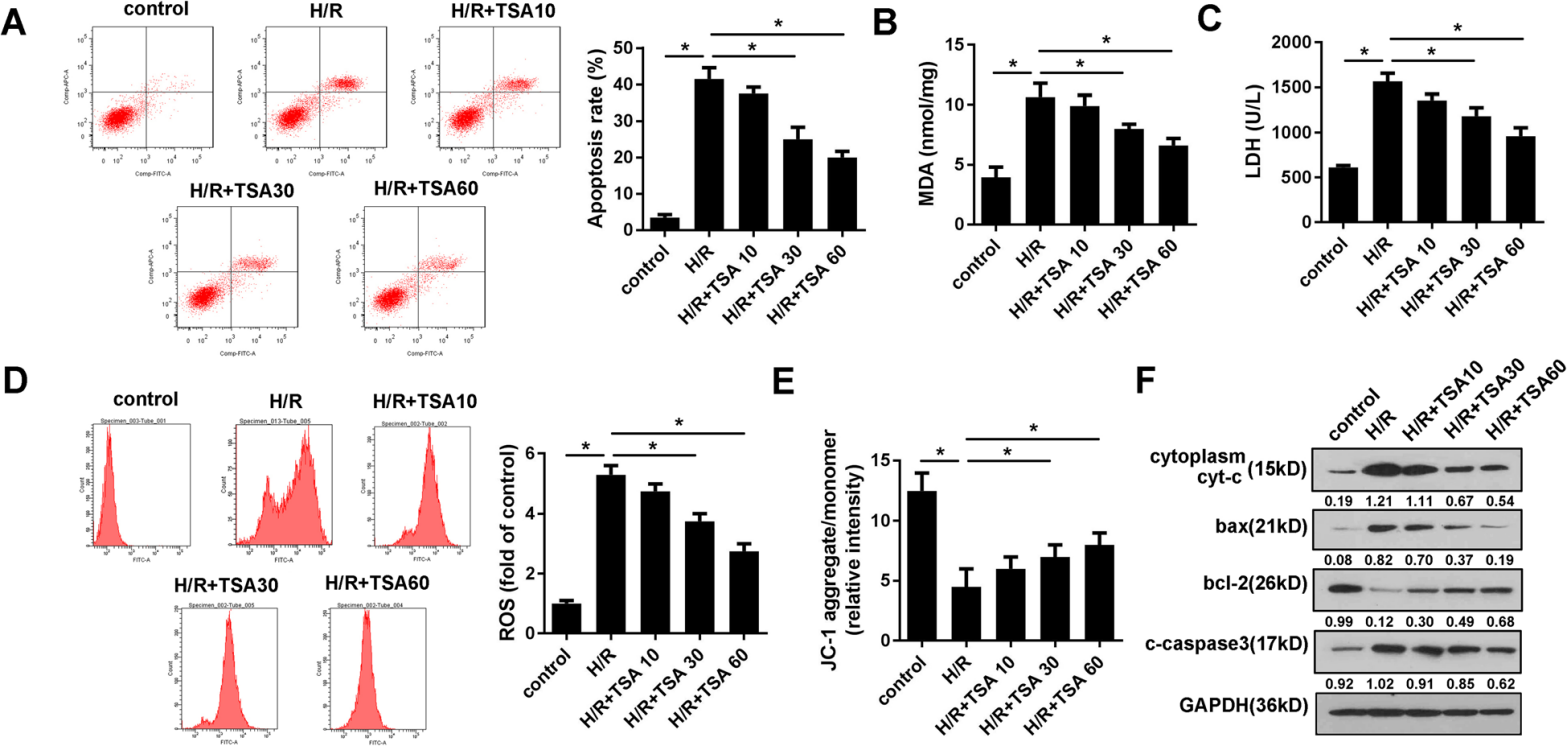
TSA alleviates apoptosis, oxidative stress, and MMP loss induced by H/R. **a** Apoptosis of cardiomyocytes was evaluated using Annexin/VI-PI staining under different dose of TSA treatment. **b**, **c** MDA and LDH amount in cardiomyocytes were analysed using commercial kit. **d** The ROS of cardiomyocytes was detected using commercial kit and flow cytometry. **e** JC-1 staining was used to evaluate the MMP. **f** Western blot was carried out to detect the apoptotic protein expression such as bax, cytoplasma cyt-c, cleaved caspase3, and bcl-2. *p < 0.05

TSA has been reported to be capable of attenuating the I/R injury in cardiomyocytes. To evaluate this function, we first established a H/R model of cardiomyocytes. As shown in Fig. 1a, TSA concentrations of 30 and 60 μM reduced the H/R induced apoptosis of cardiomyocytes. In addition, the levels of MDA, LDH, and ROS were detected to evaluate the oxidative stress of cardiomyocytes. We found that while H/R significantly elevated the levels of MDA, LDH, and ROS, TSA concentrations of 30 and 60 μM notably reduced this elevation (Fig. 1 b-d). Moreover, MMP was evaluated via JC-1 staining. As showed in Fig. 1e, TSA at 30 and 60 μM concentrations elevated MMP which was otherwise decreased by H/R treatment. In order to further verify the effect of TSA on cell apoptosis, we detected the expression level of apoptotic proteins. It was seen that H/R promoted expression levels of apoptotic proteins such as bax, cytoplasma cyt-c, and cleaved caspase3, while inhibiting the anti-apoptotic protein bcl-2. Three doses of TSA reversed the alterations induced by H/R (Fig. 1f).

### TSA promoted the expression of AK003290

**Fig. 2:**
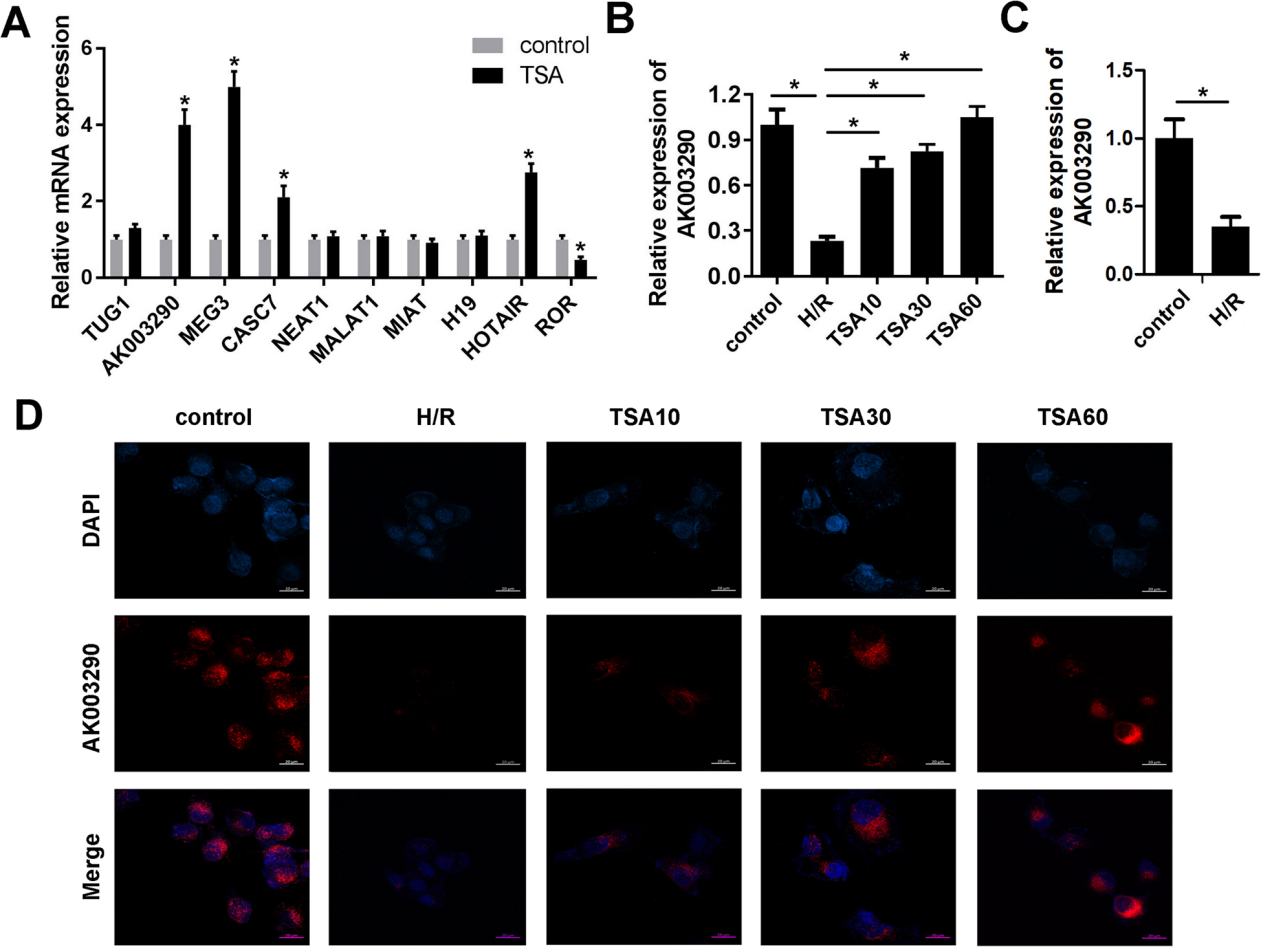
TSA promoted the expression level of AK003290. **a** qPCR was used to evaluate the expression of several lncRNAs implicated in heart disease. **b** qPCR was used to evaluate the expression of AK003290 in cardiomyocytes under different dose of TSA treatment. **c** qPCR was used to evaluate the expression of AK003290 in myocardium subjected to H/R injury. **d** FISH experiment was used to evaluate the expression of AK003290 under different dose of TSA treatment. *p < 0.05

To explore novel mechanisms underlying the effects of TSA, we focused on lncRNA which has attracted a lot of attention lately due to their diverse functions. We searched several lncRNAs that have been reported to play critical roles in cardiovascular system diseases and evaluated their expression levels after TSA treatment. The results indicated that TSA elevated the levels of AK003290, MEG3, CASC7, HOTAIR while decreased that of ROR (Fig. 2a). Furthermore, we selected and evaluated the expression of AK003290 after H/R and TSA treatment using qPCR and FISH assays. As Fig. 2b reveals, AK003290 was found to be notably downregulated by H/R treatment. However, TSA significantly reversed the effects of H/R (Fig. 2b). To confirm the effect of AK003290 in the I/R injury. We established I/R model using mice and detected the expression of AK003290 in the myocardium. As expected, AK003290 was significantly down-regulated in the myocardium subjected to H/R (Fig. 2c). Further, we performed FISH experiment. According to the qPCR results, AK003290 was found to be downregulated by H/R treatment. However, TSA significantly reversed the effects of H/R (Fig. 2d).

### Knock down of AK003290 reversed the effect of TSA on H/R injury

**Fig. 3:**
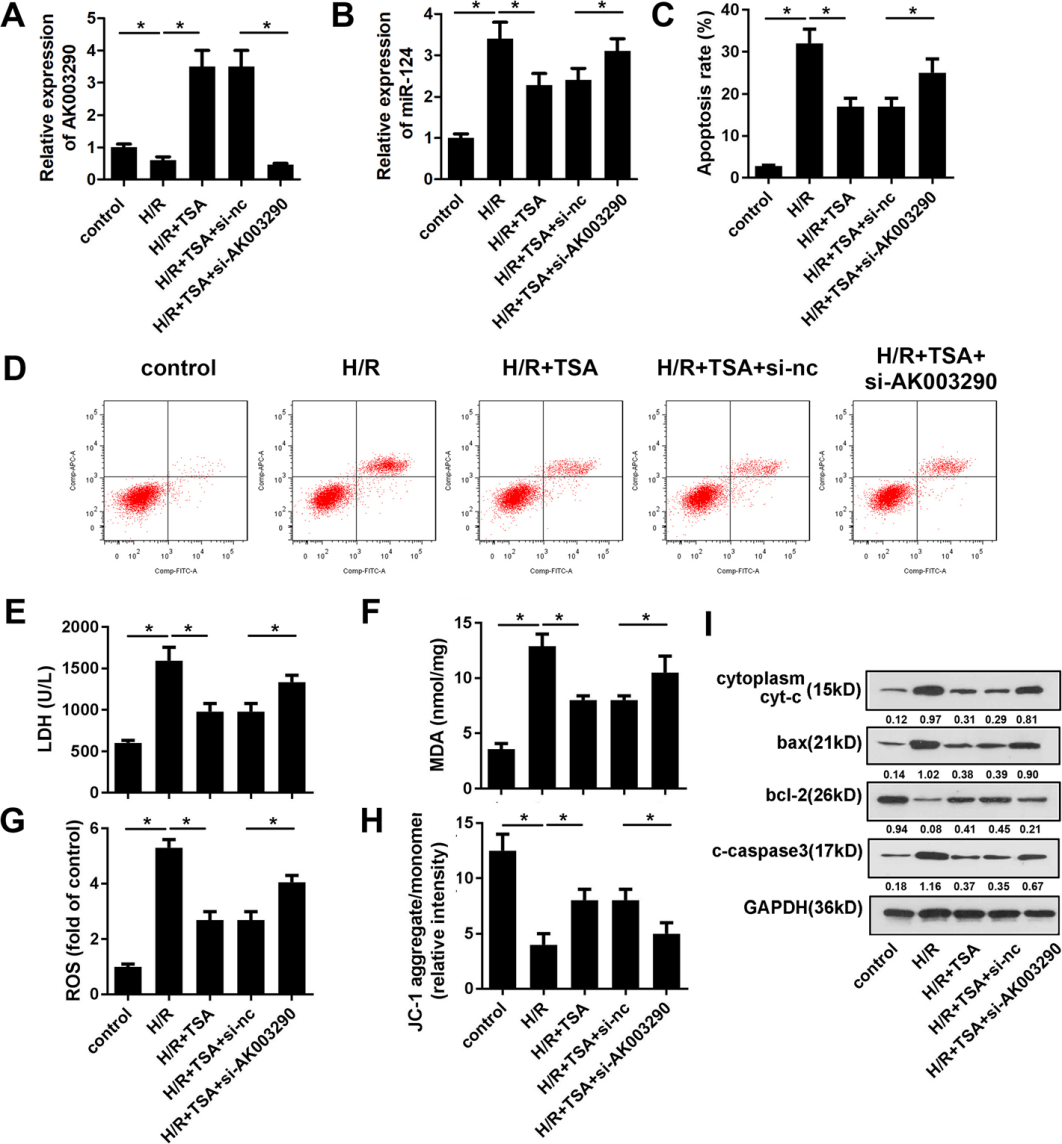
Knock down of AK003290 reversed the effect of TSA. **a, b** qPCR was performed to evaluate the expression level of AK003290 and miR-124. **c. d** Apoptosis of cardiomyocytes was evaluated using Annexin/VI-PI staining under TSA treatment and si-AK003290 transfection. **e, f** MDA and LDH amount in cardiomyocytes were analysed using commercial kit. **g** The ROS of cardiomyocytes was detected using commercial kit and flow cytometry. **h** JC-1 staining was used to evaluate the MMP. **i** Western blot was carried out to detect the apoptotic protein expression such as bax, cytoplasma cyt-c, cleaved caspase3, and bcl-2. *p < 0.05

As AK003290 downregulated by H/R was found to be upregulated by TSA treatment, we speculated that AK0032090 exerts a protective role against H/R injury in cardiomyocytes. To determine this, we knocked-down AK003290 using an siRNA. First, we detected the expression of AK003290 and miR-124-5p in different groups. The results indicated that H/R notably inhibited the level of AK003290, TSA treatment obviously promoted that while si-AK003290 again decreased it (Fig. 3a). In addition, H/R promoted the expression of miR-124 while TSA decreased it. Si-AK003290 elevated the miR-124 level notably (Fig. 3b). The knock down of AK003290 reversed the effect of TSA (30 μM) on apoptosis (Fig. 3c, d). Moreover, the same trend was found during LDH, MDA, oxidative stress and MMP loss (Fig. 3e-h). We found that AK003290 knock down increased the expression level of bax, cytoplasma cyt-c, cleaved caspase3 and reduced that of bcl-2 in cardiomyocytes compared to TSA treatment group (Fig. 3i).

### AK003290 sponges miR-124-5p in cardiomyocytes

**Fig. 4:**
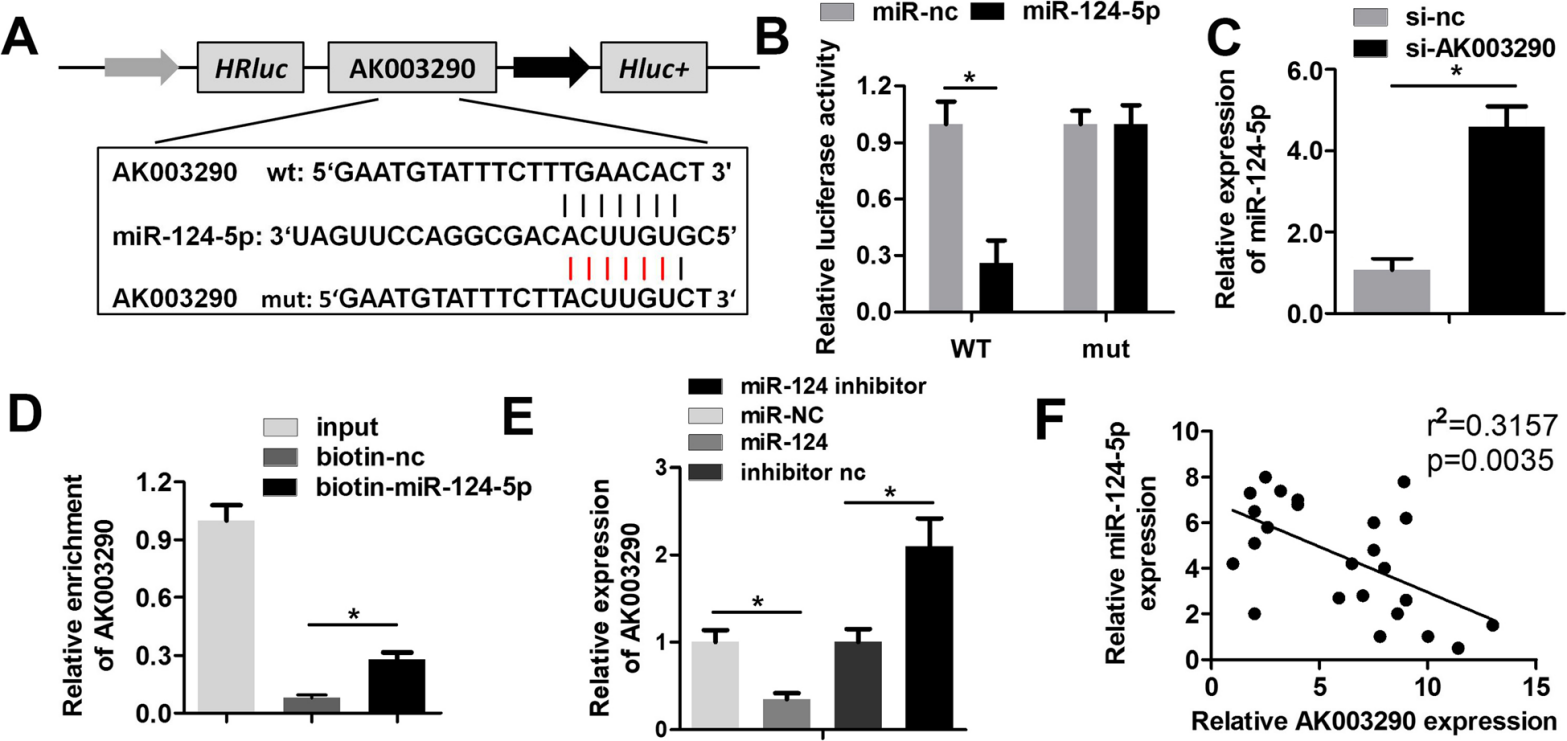
MiR-124-5p directly targets AK003290 in cardiomyocytes. **a** The targeting region between miR-124-5p and AK003290. **b** Luciferase activity assay was carried out to verify whether miR-124-5p targets AK003290 in cardiomyocytes. **c** qPCR was performed to detect the expression of miR-124-5p. **d** RNA pull down using the probe of miR-124-5p was performed to detect the interaction between miR-124-5p and AK003290. **e** qPCR was performed to detect the expression of AK003290. **f** Pearson analysis was performed to investigate the correlation between circCadm1 and Pawr as well as miR-124-5p targets AK003290. *p < 0.05

LncRNAs are capable of ‘sponging’ miRNAs to block their regulatory function on target genes. Here, we predicted and verified the miRNA targeted by AK003290 to further elucidate the mechanism underlying the effect of this lncRNA. Figure 4a shows the targeting sequences between AK003290 and miR-124-5p. Luciferase assay indicated that miR-124-5p is directly targeted by AK003290 in cardiomyocyte (Fig. 4b). Furthermore, the level of miR-124-5p was notably reduced in AK003290 knock down group (Fig. 4c). RNA pull-down was carried out to detect whether AK003290 could directly bind to miR-124-5p endogenously. We found that biotin labelled miR-124-5p probe enriched the level of AK003290, that is indicative of an interaction between AK003290 and miR-124-5p (Fig. 4d). Moreover, the qPCR analysis further confirmed that miR-124-5p overexpression decreased the level of AK003290, while knock down of miR-124-5p increased that of AK003290 (Fig. 4e). Lastly, Pearson analysis was carried out which indicated a negative correlation between miR-124-5p and AK003290 (Fig. 4f).

### MiR-124-5p overexpression reversed the effect of TSA on H/R injury

**Fig. 5:**
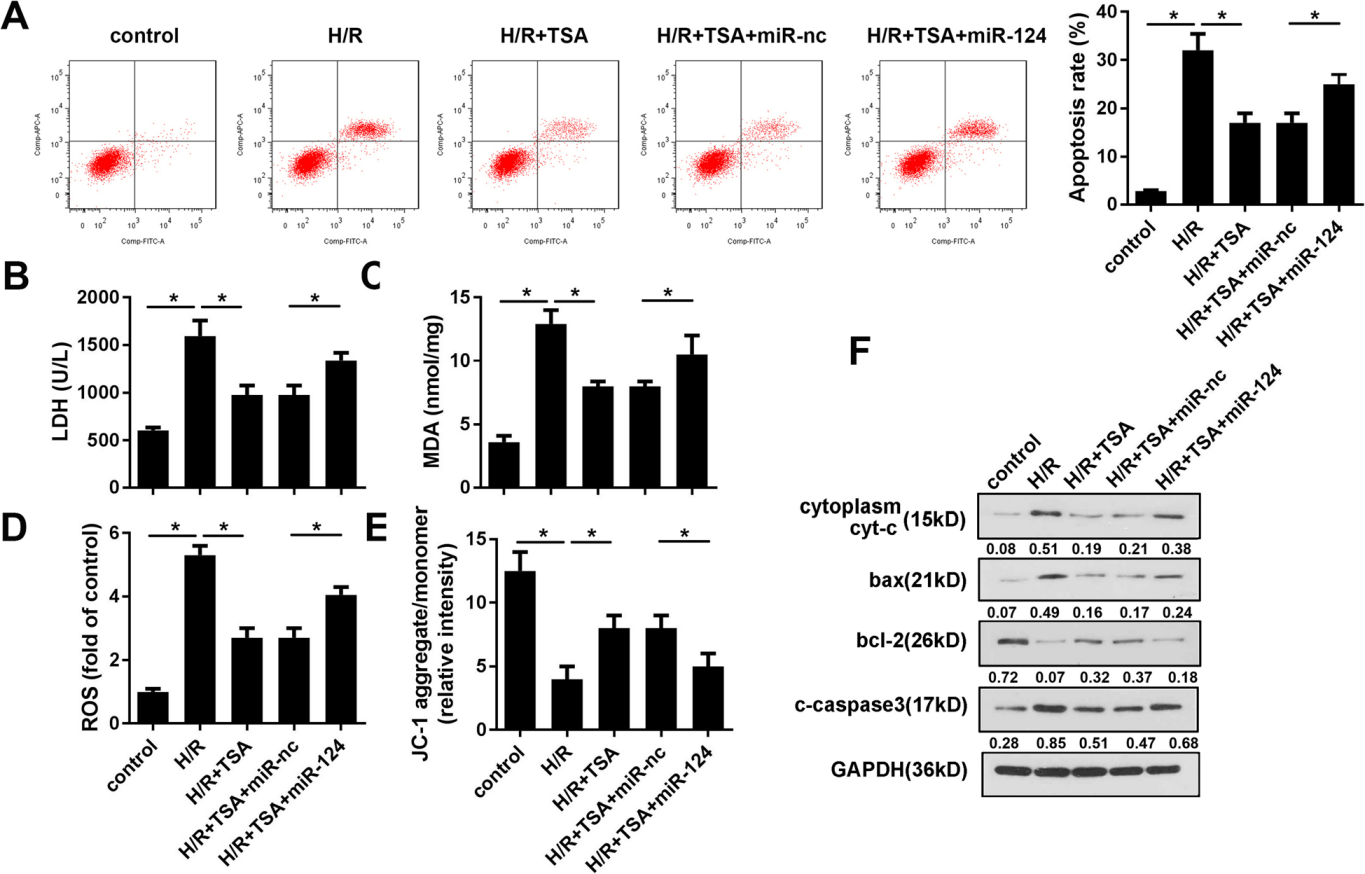
MiR-124-5p overexpression reversed the effect of TSA. **a** Apoptosis of cardiomyocytes was evaluated using Annexin/VI-PI staining under TSA treatment and miR-124-5p mimic transfection. **b, c** MDA and LDH amounts in cardiomyocytes were analysed using commercial kit. **d** The ROS of cardiomyocytes was detected using commercial kit and flow cytometry. **e** JC-1 staining was used to evaluate the MMP. **f** Western blot was carried out to detect the apoptotic protein expression such as bax, cytoplasma cyt-c, cleaved caspase3, and bcl-2. *p < 0.05

To confirm whether miR-124-5p participates in the function of TSA on H/R injury, rescue experiment was carried out. It was found that miR-124-5p overexpression reversed the effect of TSA on apoptosis (Fig. 5a). Moreover, the same trend was found during the oxidative stress (Fig. 5b-d) and MMP loss (Fig. 5e). We further looked at relation between miR-124-5p and apoptosis and found that miR-124-5p overexpression increased the expression levels of bax, cytoplasma cyt-c and cleaved caspase3, while reducing the level of bcl-2 of cardiomyocytes in comparison to TSA treatment group (Fig. 5f).

### MiR-124-5p knock down reversed the effect of si-AK003290 on H/R injury

**Fig. 6:**
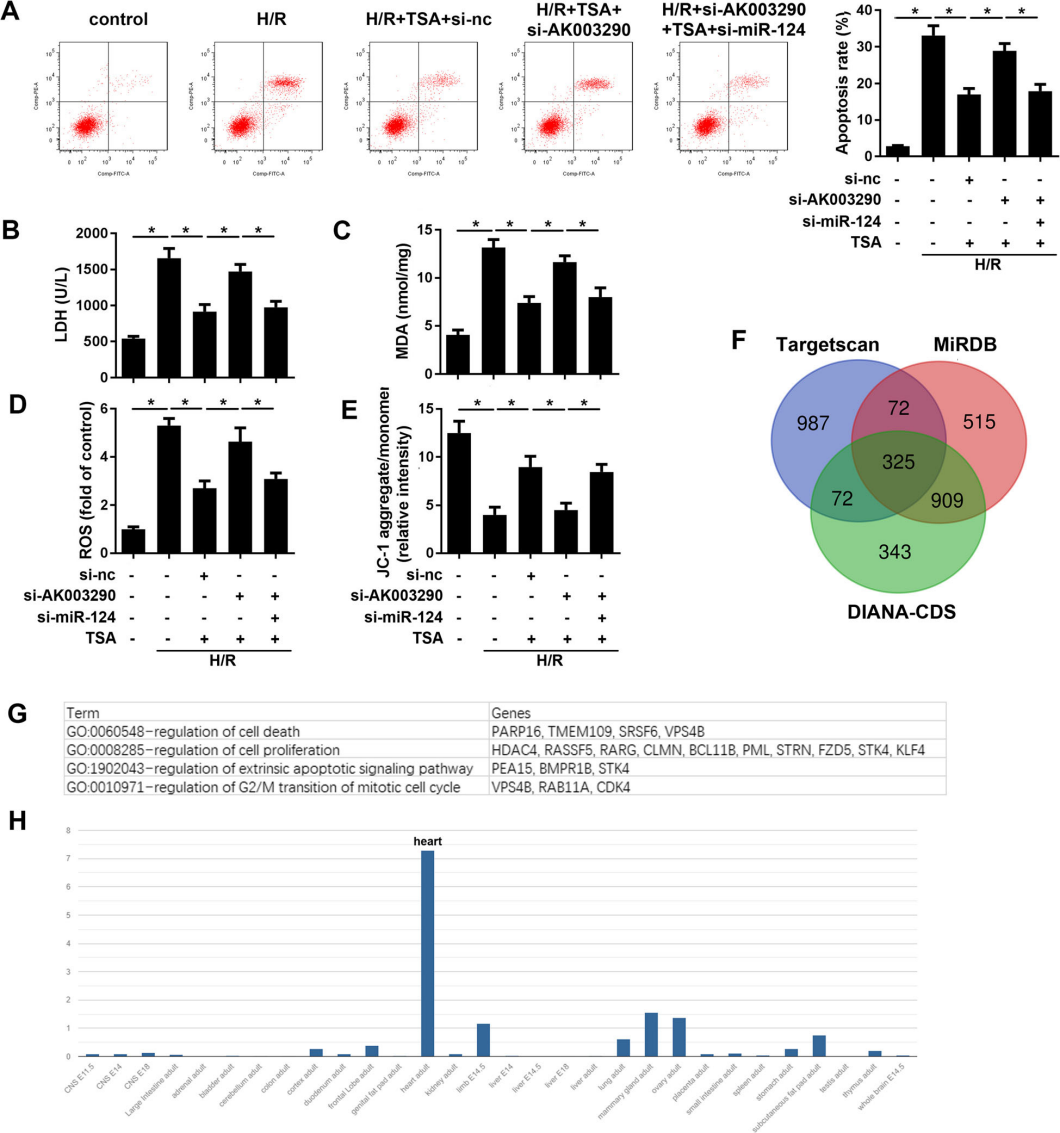
MiR-124-5p knock down reversed the effect of si-AK003290. **a** Apoptosis of cardiomyocytes was evaluated using Annexin/VI-PI staining under TSA treatment and miR-124-5p mimic transfection. **b, c** MDA and LDH amounts in cardiomyocytes were analysed using commercial kit. **d** The ROS of cardiomyocytes was detected using commercial kit and flow cytometry. **e** JC-1 staining was used to evaluate the MMP. **f** Targetscan, DIANA-CDS and miRDB were used to predicted the targets of miR-124. Venn analysis was performed to take intersection of the three set. **g** David analysis was carried out to analyze and enrich those target genes. We displaced the genes related to survival or apoptosis. **h** The distribution of AK003290 in different organs was showed. *p < 0.05

To confirm whether miR-124-5p participates in the function of AK003290 on H/R injury, rescue experiment was carried out. It was found that miR-124-5p knock down reversed the effect of si-AK003290 on apoptosis (Fig. 5a). Moreover, the same trend was found during the oxidative stress (Fig. 5b-d) and MMP loss (Fig. 5e). It is well known that miRs inhibit the expression of their target genes. To elucidate the molecular mechanism, we predicted the potential tartes of miR-124 using targetscan, DIANA-CDS and miRDB software and obtained 325 genes exist in all the three date sets (Fig. 6f). We enriched these genes using David software, and placed some genes that are related to cell proliferation or apoptosis which will be studied in the future (Fig. 6g). We further analyzed the organ-specific expression of AK003290 in mice. As revealed in Fig. 6h, AK003290 is enriched in the heart of mice. This result indicated the critical role of AK003290 in the progression of cardiac vascular diseases.

## Discussion

Apoptosis is well known to critically cause myocardial cell death induced by dysfunction of mitochondria and increase of lipid peroxides mainly due to I/R injury. Deeper understanding of the mechanisms of cardiomyocyte apoptosis is critical to prevent heart injury and treat heart disease. In the present study, we detected apoptosis of cardiomyocytes using flow cytometry and to further evaluate the apoptotic condition, we detected the levels of MMP and apoptotic proteins including bax, cytoplasm cyt-c, bcl-2 and cleaved caspase3. It was found that TSA reduced the frequent occurrence of apoptosis of cardiomyocytes and alleviated the oxidative stress and MMP loss induced by H/R treatment.

TSA has been extensively studied not only in cardiovascular I/R injury, but also in cerebral I/R injury. These findings proved the protective function of TSA on myocardial I/R injury to a great extent. However, the molecular mechanism underlying the mechanism of action of TSA is extremely complicated. It was reported that TSA participates in the regulation of classic signaling pathways such as the AMPKs/mTOR [16], PI3K/Akt/FOXO3A/Bim [17], MAPK [18] pathways as well as some key proteins including TORC1, pCREB, BDNF [19], and Na+/K + -ATPase [20]. However, the interaction between TSA and lncRNAs has been rarely studied.

Emerging evidences have shown that non-coding RNAs such as miRNAs and lncRNAs possesses crucial roles in many biological processes. Previously, TSA was found to be capable of inhibiting miR-1 expression through p38 MAPK signal pathway in post-infarction rat cardiomyocytes [18]. In the present study, we identified that lncRNA AK003290 was downregulated in cardiomyocytes subjected to H/R, which is indicative of its protective role against H/R injury. Interestingly, TSA treatment elevated the level of AK003290. We speculated that this is because AK003290 is involved in the effect of TSA on reversing H/R injury. Further experiments revealed that knock down of AK003290 notably reversed the effect of TSA on apoptosis, oxidative stress and loss of MMP. These findings verified that TSA protected cardiomyocytes from H/R induced injury via up-regulating AK003290 expression.

It is well known that lncRNAs always act as sponges of miRNA, and thus participate in the regulation of their target genes involved in common cellular functions. Previous studies have reported that APF, MALAT1, and PFL mediate I/R injury through sponging miR-188-3p, miR-155/miR-203/miR-133, and let-7d respectively. Therefore, to further understand the critical role of AK003290 in cardiac I/R injury, we further explored the molecular mechanism by which they exert their functions. Bioinformatic tools predicted that AK003290 is a potential target of miR-124-5p, and luciferase assay determined that AK003290 acts as a direct target of miR-124-5p, as we speculated. Finally, rescue experiment further confirmed our speculation by identifying miR-124-5p as a downstream gene regulated by TSA. This is the first report demonstrating the function of TSA through lncRNA/miR signaling.

MiR-124-5p is a rarely studied miRNA. It is known from previous studies that miR-124-5p inhibits the growth of high-grade gliomas through posttranscriptional regulation of LAMB1 [21]. Additionally, low expression levels of miR-124-5p correlated with poor prognosis in colorectal cancer via targeting of SMC4. Here, we confirmed that miR-124-5p directly targets AK003290. We also performed rescue experiments and found both AK003290 knock down and miR-124-5p overexpression can reverse the effect of TSA. Rescue experiment further confirmed that AK003290 exerts its role in H/R injury via modulating miR-124 expression.

It is well known that miRs modulate the expression of their target genes. Here, we predicted the potential targets of miR-124 using three prediction software. Then, we enriched these gene into specific biological process using David software. These genes that are involved in cell proliferation and apoptosis will be studied in the future.

## Conclusion

In conclusion, we extended the understanding of the role of lncRNA in cardiac ischemia/reperfusion injury and provided a novel regulatory mechanism underlying the effect of TSA that is AK003290/miR-124-5p signaling. Our findings suggest novel biomarkers or potential therapeutic targets to treat ischemic heart diseases.

## Methods

### Cell culture

H9c2 cell (catalog: GNR 5) were obtained from Cell bank of Chinese Academy of Sciences and cultured in DMEM (Gibco, CA, USA) supplemented with 10% fetal bovine serum (FBS, Gibco, CA, USA), 100 U/mL penicillin and 100 μg/mL streptomycin under 5% CO_2_ at 37 °C in a humidified atmosphere.

### I/R model establishment

20 12-week-old female C57BL/6 mice were obtained from Charles River Laboratories and maintained in the specific pathogen free (SPF) environment with free access to water and food. The mice were randomly divided into two groups including sham and I/R group. All animal procedures were performed in accordance with institutional guidelines and approved by the Animal Studies Committee of Health Science Center, Peking University. The left thoracotomy was performed to expose the heart. Then, the left coronary artery (LCA) was ligated using a 6–0 silk suture. After occlusion for 30 mins, blood supply was restored for 2 h by loosening the suture. After 2 h reperfusion, the rats were sacrificed and the hearts were harvested. All rats underwent the same I/R procedure, while sham group experienced the surgical procedure without the ligation of left anterior descending coronary artery (LAD). After reperfusion, mice were anaesthetized by injection with 1% pentobarbital sodium (40 mg/kg) intraperitoneally, .20 heart tissues come from the sham and I/R group were extracted for the further research.

### Drug treatment

TSA were purchased from National Institute for the Control of Pharmaceutical and Biological Products (> 99% purity, Beijing, China). Cells were treated with TSA (0.5, 1, 5 μM) respectively 2 h before inducing the hypoxia and during the hypoxia period.

### Plasmids construction

MiR-124-5p mimic and mimic control as well as siRNA for AK003290 and the negative control were designed and synthesized by Genepharma (Shanghai, China). The wild and mutant region of AK003290 that was to be targeted by miR-124-5p were synthesized by Genepharma (Shanghai, China) and cloned into pGL3 luciferase reporter vectors (Promega, CA, USA).

### Flow cytometry

The cultured H9c2 cells were digested with trypsin, washed with cold PBS and dual-stained with Annexin V-FITC/propidium iodide according to the manufacturer’s instructions. Cell apoptosis was detected by flow cytometry on a BD FACSCalibur (Becton Dickinson, NJ, USA).

### Reverse transcription and quantitative realtime PCR

RNA extraction was performed with Trizol reagent (Invitrogen, CA, USA), precipitated with isopropanol, washed with 75% ethanol and dissolved in RNase free water. Reverse transcription was performed with 1 μg RNA using the cDNA transcription kit (Transgen, USA). 20 ng cDNA was used for qPCR to validate the expression of relative mRNA. It was determined by using SYBR green mix (Yisheng, Shanghai, China).

### Western blotting

Cells were lysed on ice using a lysis buffer (Beyotime, Shanghai, China). The proteins were separated by centrifuging at 12000 g for 10 min at 4 °C. Protein lysates were loaded with 5× loading buffer on the SDS-PAGE. After electrophoresis, the gel was transferred to PVDF membrane. Followed by blocking with skim milk for 1 h, protein bands on the PVDF membrane were incubated with relative primary antibodies at 4 °C overnight and the corresponding secondary HRP antibody at room temperature for 2 h. The blots were visualised by ECL chemiluminescence.

### RNA pull-down assay

Biotinylated miR-124-5p probe and the control probe were synthesized by Sangon Biotech (Shanghai, China). Probe-coated beads were generated by co-incubating the probe with streptavidin-coated beads (Invitrogen, CA, USA) at 25 °C for 2 h. H9c2 cells were collected, lysed, and incubated with AK003290 or miR-124-5p probes overnight at 4 °C. Thereafter, the beads were eluted, and the complex was purified with TRIzol (Takara, Dalian, China). Then, the abundance of AK003290 and miR-124-5p was analyzed by qRT-PCR.

### LDH and MDA detection

After reperfusion, the hearts were resected and homogenized in cold phosphate buffer. After centrifuging at 3000 rpm for 15 min, the supernatant was collected for measurement at − 20 °C. The levels of LDH as well as MDA were evaluated using commercial kits following manufacturer’s protocols (Jiancheng, Nanjing, China).

### ROS detection

After I/R treatment, myocardial tissue or cardiomyocytes were collected and washed in PBS followed by co-culturing with 10 μM DCFH-DA (DCF-DA, Shanghai, Beyotime) at 37 °C for 20 min with gentle shaking in the dark. The mean fluorescence intensity (MFI) was evaluated using a flow cytometer (BD FACSCalibur, Becton Dickinson, NJ, USA).

### JC-1 staining

After treatment, the transfected cells were incubated with 10 mM 5, 5′,6, 6′-tetrachloro-1, 1′,3, 3′- tetraethylbenzimidazolylcarbocyanine iodide (JC-1) (Beyotime, Shanghai, China) for 30 min at 37 °C. Then, the fluorescence labeled cells were washed with PBS and analyzed by GraphPad Prism software. The ratio of fluorescence at 590 nm versus 530 nm emission was applied to measure the mitochondrial membrane potential (MMP).

### Fluorescence in situ hybridization (FISH)

Alexa Fluor 555-labelled AK003290 probes were designed and synthesized by RiboBio (Guangzhou, China). The experiment was carried out with a Fluorescent In Situ Hybridisation kit (RiboBio, Guangzhou, China). The cells were seeded onto autoclaved glass slides at a density of 1 × 105 cells and cultured for 24 h. After fixing with 4% paraformaldehyde for 20 min followed by permeabilization with 0.5% Triton X-100 for 10 min, the cells were cultured at 37 °C overnight. Finally, the slides containing cells were incubated with DAPI to stain the cell nucleus and observed under a fluorescence microscope (Leica, Wetzlar, Germany).

### Luciferase assay

The wild-type (WT) or mutant (mut) seed sequence at the predicted region in AK003290 and miR-124-5p were synthesized and cloned into the pGL3 Luciferase Reporter Vectors (Promega, CA, USA) at the KpnI and BamHI sites. H9c2 cells were co-transfected with either miR-124-5p mimic or mimic control, together with pGL3 vectors, which contained the WT or mut predicted binding regions of AK003290. TRL-SV40 plasmid (Promega, CA, USA) was also transfected as a normalizing control. The cells were harvested to detect of luciferase activity using Dual-Luciferase Assay kit (Promega, WI, USA) 48 h post-transfection.

### Statistical analysis

SPSS 20.0 software (SPSS Inc., Chicago, IL, USA) were used to analyze all data for statistical significance. All the data are presented as the means ± SD. One-way ANOVA was used to assess the difference between multiple groups. Differences between two groups were analyzed by the Student’s t-test. *P* < 0.05 was considered as statistical significance.

## Data Availability

All data and materials are available in the manuscript and figures.

## Abbreviations

AMI: Acute myocardial infarction
TSA: Tanshinone IIA
qRT-PCR: Quantitative real-time PCR
MMP: Mitochondrial membrane potential
H/R: Hypoxia/reoxygenation
I/R: Ischemia/reoxygenation
SPF: Specific pathogen free
FBS: Fetal bovine serum
LCA: Left coronary artery
LAD: Left anterior descending coronary artery
JC-1: 5, 5′,6, 6′-tetrachloro-1, 1′,3, 3′- tetraethylbenzimidazolylcarbocyanine iodide
FISH: Fluorescence in situ hybridization
WT: Wild-type
mut: Mutant
